# Increased infiltration of CD4^+^ T cell in the complement deficient lymphedema model

**DOI:** 10.1186/s12865-023-00580-1

**Published:** 2023-11-08

**Authors:** Toshihiko Nishioka, Kei-ichi Katayama, Shinji Kumegawa, Kyoichi Isono, Takashi Baba, Hiroshi Tsujimoto, Gen Yamada, Norimitsu Inoue, Shinichi Asamura

**Affiliations:** 1 Department of Plastic Surgery, Wakayama Medical University, 811-1 Kimiidera, Wakayama, 641-8509 Japan; 2 Department of Molecular Genetics, Wakayama Medical University, 811-1 Kimiidera, Wakayama, 641-8509 Japan; 3 Laboratory Animal Center, Wakayama Medical University, 811-1 Kimiidera, Wakayama, 641-8509 Japan

**Keywords:** Lymphedema, Complement, C3, CD4^+^ T cell, Dead cells

## Abstract

**Background:**

Lymphedema is an intractable disease that can be caused by injury to lymphatic vessels, such as by surgical treatments for cancer. It can lead to impaired joint mobility in the extremities and reduced quality of life. Chronic inflammation due to infiltration of various immune cells in an area of lymphedema is thought to lead to local fibrosis, but the molecular pathogenesis of lymphedema remains unclear. Development of effective therapies requires elucidation of the immunological mechanisms involved in the progression of lymphedema. The complement system is part of the innate immune system which has a central role in the elimination of invading microbes and acts as a scavenger of altered host cells, such as apoptotic and necrotic cells and cellular debris. Complement-targeted therapies have recently been clinically applied to various diseases caused by complement overactivation. In this context, we aimed to determine whether complement activation is involved in the development of lymphedema.

**Results:**

Our mouse tail lymphedema models showed increased expression of C3, and that the classical or lectin pathway was locally activated. Complement activation was suggested to be involved in the progression of lymphedema. In comparison of the *C3* knockout (KO) mouse lymphedema model and wild-type mice, there was no difference in the degree of edema at three weeks postoperatively, but the *C3* KO mice had a significant increase of TUNEL^+^ necrotic cells and CD4^+^ T cells. Infiltration of macrophages and granulocytes was not significantly elevated in *C3* KO or *C5* KO mice compared with in wild-type mice. Impaired opsonization and decreased migration of macrophages and granulocytes due to C3 deficiency should therefore induce the accumulation of dead cells and may lead to increased infiltration of CD4^+^ T cells.

**Conclusions:**

Vigilance for exacerbation of lymphedema is necessary when surgical treatments have the potential to injure lymphatic vessels in patients undergoing complement-targeted therapies or with complement deficiency. Future studies should aim to elucidate the molecular mechanism of CD4^+^ T cell infiltration by accumulated dead cells.

**Supplementary Information:**

The online version contains supplementary material available at 10.1186/s12865-023-00580-1.

## Background

Lymphedema is characterized by abnormal accumulation of lymphatic fluid resulting in intractable and progressive tissue swelling [1, 2]. Formation of lymphedema often interferes with joint mobility in the extremities, which can negatively impact upon a patient’s quality of life. Postoperative lymphedema is not uncommon in patients treated for malignant tumors such as breast, gynecological, and genitourinary cancers [3]. Surgical treatments, including lymph node dissection, skin excision and radiation therapy, can cause damage to lymphatic vessels, leading to abnormal formation of collateral lymphatic vessels and impaired lymphatic function [2, 4, 5]. Lymphedema has virtually no effective treatments [6], so to prevent disease progression or to ameliorate symptoms there are only conservative treatments consisting of multimodal approaches known as ‘complete decongestive therapy’ [7–10].

In the pathophysiology of lymphedema, inflammation plays an essential role in both experimental and clinical studies [11]. Inflammatory cells infiltrate lymphedema tissue such as macrophages, neutrophils, and lymphocytes [12]. Infiltration of CD4^+^ T cells was suggested in a mouse model to be involved in fibrosis and impaired function of lymphatic vessels [13]. However, there are insufficient reports on the molecular mechanisms by which immune cells infiltrate lymphedema tissue. The wound healing process is also known to be accompanied by infiltration of many immune cells and fibrosis. Expression of genes related to acute inflammation and wound healing was also reportedly upregulated in the tissue of a mouse lymphedema model [14].

The complement system functions as an innate immune system which recognizes pathogens and altered host cells, protecting the host from them and maintaining homeostasis. It is also known to be activated in the wound healing process [15, 16]. In a mouse model, the deficiencies of complement components C3, C5 and the C5a receptor 1 (C5aR1) accelerated the process of cutaneous wound healing [17]. The accelerated healing has been suggested to be mediated by the inhibition of C5aR1 signaling and the decreased infiltration of inflammatory cells to wound regions. However, the roles of the complement system in formation of lymphedema remain unclear. All three complement activation pathways in the complement system [the classical pathway, the lectin pathway, and the alternative pathway (AP)] act to form the C3 convertase complex, which degrades C3, generating C3a and C3b [18]. The terminal pathway is initiated through the cleavage of the 5th component of complement, while C5a, the 9 kDa cleavage product acts as a potent anaphylatoxin and chemotactic reagent major, C5b the major cleavage product C5 initiates the formation of membrane attack complex (MAC) through subsequent binding of the terminal pathway components C6, C7, C8 to form the C5b-C8 complex that inserts into the bilayer of cell membranes and initiates the binding, polymerization and insertion of poly-C9 which forms a cylindrical pore that penetrates the cellular membrane and poses an osmolytic challenge to target cells coated with MAC. The major cleavage product of complement C3 cleavage, C3b can either bind to the AP zymogen factor B to form the AP zymogen complex C3bB or bind covalently to the pathogen surfaces where it is cleaved into its degradation product iC3b or C3dg to serve as ligands of C3-receptors CR1, CR2, CR3 and CR4 or CD18 (which belongs to the family of β-integrins) [18, 19].

Eculizumab and ravulizumab, humanized monoclonal antibodies blocking cleavage of C5, have been clinically applied to two disorders, paroxysmal nocturnal hemoglobinuria (PNH) and atypical hemolytic uremic syndrome, in which activation of the complement system by abnormal complement regulatory mechanisms destroys autologous cells [18, 20]. These antibodies have also been used in treatment of neurological disorders caused by autoantibodies such as myasthenia gravis and neuromyelitis optica spectrum disorders [21]. If complement activation was shown to have a significant effect on the development of lymphedema, these treatments could be expected to be effective in treating lymphedema. Meanwhile, if complement activation promoted the development of lymphedema, the potential for administration of these treatments exacerbating the lymphedema should be regarded with caution. In the present study, we analyzed whether complement activation is involved in the development of lymphedema using *C3* knockout (KO) mice and *C5* KO mice.

## Results

### Upregulation and activation of C3 in the mouse tail lymphedema

**Fig. 1:**
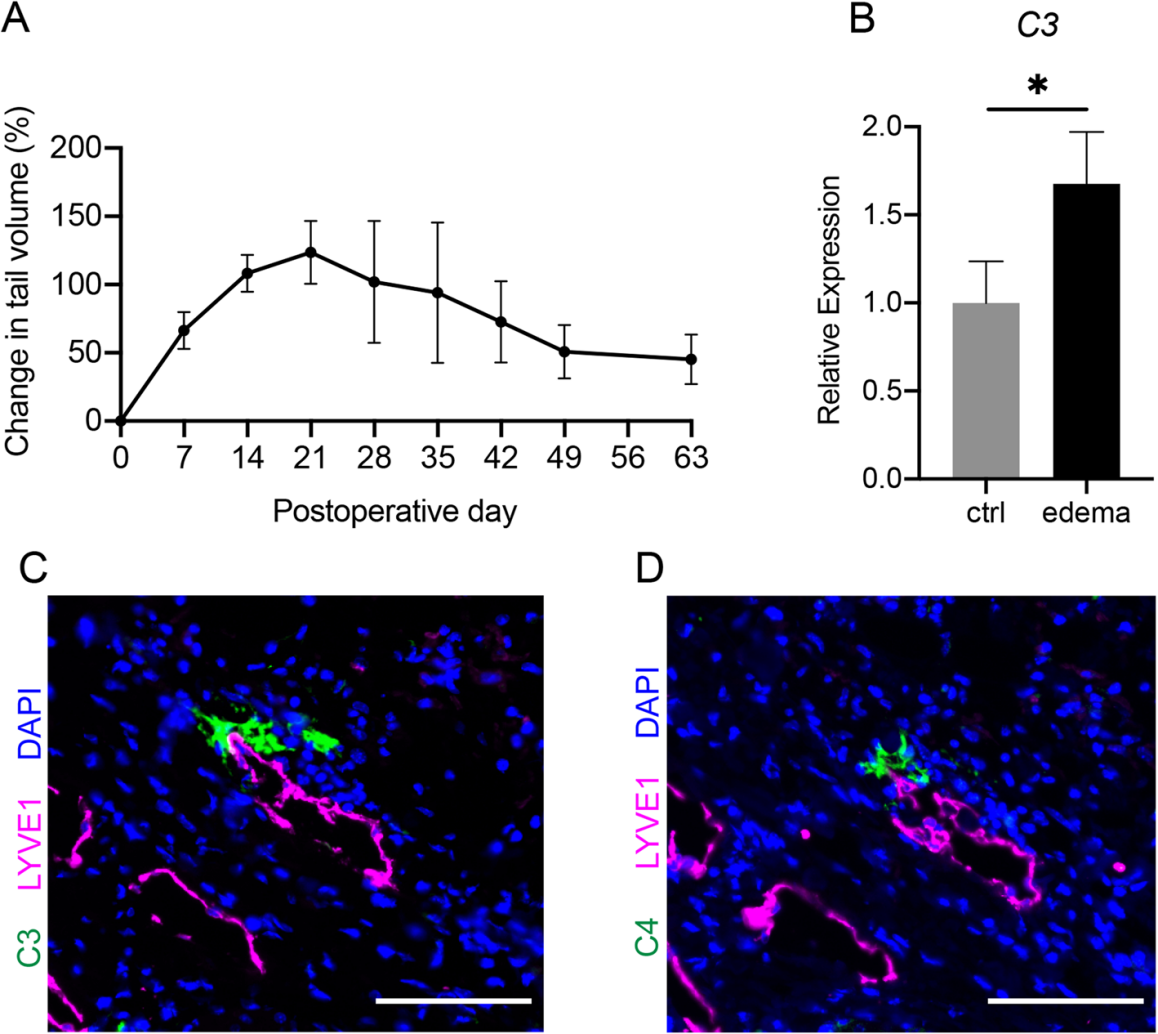
Postoperative upregulated C3 expression and complement activation in the tail lymphedema. **A** The time course of the increased ratio of tail swelling volume after the surgery in wild-type C57BL6/J  mice (*n*=3). Data are shown as the means ± standard error. **B** Relative expression levels of C3 mRNA in the tail lymphedema tissue (edema) compared with non-operated controls (Ctrl) (*n* = 3/group). The expression levels were normalized to those of 18s rRNA. Data are shown as the means ± standard deviation; *P<0.05. **C** Representative image of C3 protein (Green) around lymphatic vessels (LYVE1, Magenta) on POD21. Nuclei were stained with DAPI (Blue). Scale bars = 100 µm. **D** Representative image of C4 protein (Green) around lymphatic vessels (LYVE1, Magenta) at an adjacent section of (**C**). Nuclei were stained with DAPI (Blue). Scale bars = 100 µm

Mouse tail models have been widely used to study the cellular and molecular mechanisms of lymphedema [1, 22, 23]. In this tail model, we observed the degree of tail lymphedema. As in previous reports, prominent tail swelling reached its maximum around postoperative day (POD) 21 and then decreased gradually until entering a plateau phase around approximately POD49 (Fig. 1A) [1].

To examine the activation of the complement system in lymphedema formation, we analyzed the expression of C3 in the tissue of tail lymphedema on POD21 using real-time RT-PCR and fluorescent immunohistochemistry. Augmented expression of C3 transcript was detected in the tissue of tail lymphedema (Fig. 1B). In addition, C3 proteins were distributed around the dilated lymphatic vessels in lymphedema tissue (Fig. 1C). To further explore whether the complement system is activated in lymphedema tissue, we examined whether complement component C4, which is activated by the classical or lectin pathway, was detected at serial sections. C4 was co-localized with C3 around the dilated lymphatic vessels, suggesting that the classical or lectin pathway should be activated in the lymphedema region of the mouse tail model (Fig. 1D).

### Contribution of C3 and C5 in the mouse tail lymphedema models

**Fig. 2:**
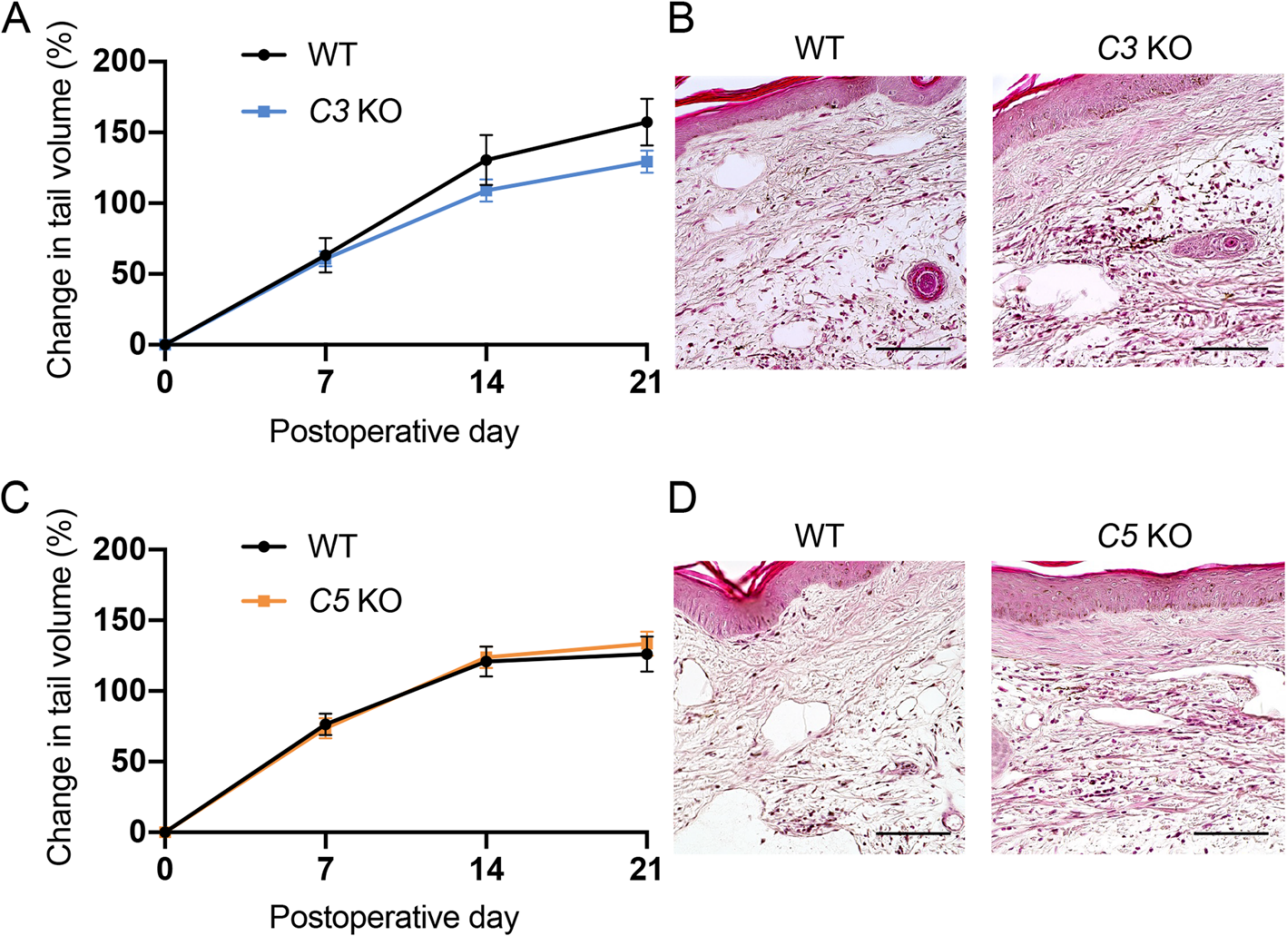
Development of lymphedema in *C3* KO and *C5* KO mice. **A** The time course of the increased ratio of tail swelling volume after the surgery in *C3* KO mice (blue line) and wild-type mice (WT, black line) (*n* = 7/group). Data are shown as means ± standard error. **B** Representative image of histology stained with H&E in wild-type (WT) and *C3* KO mice on POD21. Scale bars = 100 μm. **C** The time course of the increased ratio of tail swelling volume after the surgery in *C5* KO mice (orange line, *n* = 9) and wild-type mice (WT, black line, *n* = 7). Data are shown as means ± standard error. **D** Representative image of histology stained with H&E in wild-type (WT) and *C5* KO mice on POD21. Scale bars = 100 μm

Activation of the complement pathways was observed in lymphedema tissue, so we compared the extent of lymphedema swelling in *C3* KO mice and *C5* KO mice with that in wild-type mice. There were no significant differences in the extent of tail swelling at any of the analyzed time points (Fig. 2A and C, Supplementary Fig. 3). Next, we histologically analyzed lymphedema tissues on POD21 in *C3* KO and *C5* KO mice. More cells, including immune cells, accumulated in lymphedema tissues of *C3* KO and *C5* KO mice than in wild-type mice (Fig. 2B and D). These results suggest that inflammation is more strongly induced in lymphedema tissues of *C3* KO and *C5* KO mice compared with those of wild-type mice. We therefore used fluorescent immunohistochemistry to further examine what kind of cells are increased.

### Increased infiltration of CD4^+^T cells in lymphedema of ***C3 ***KO mice

**Fig. 3:**
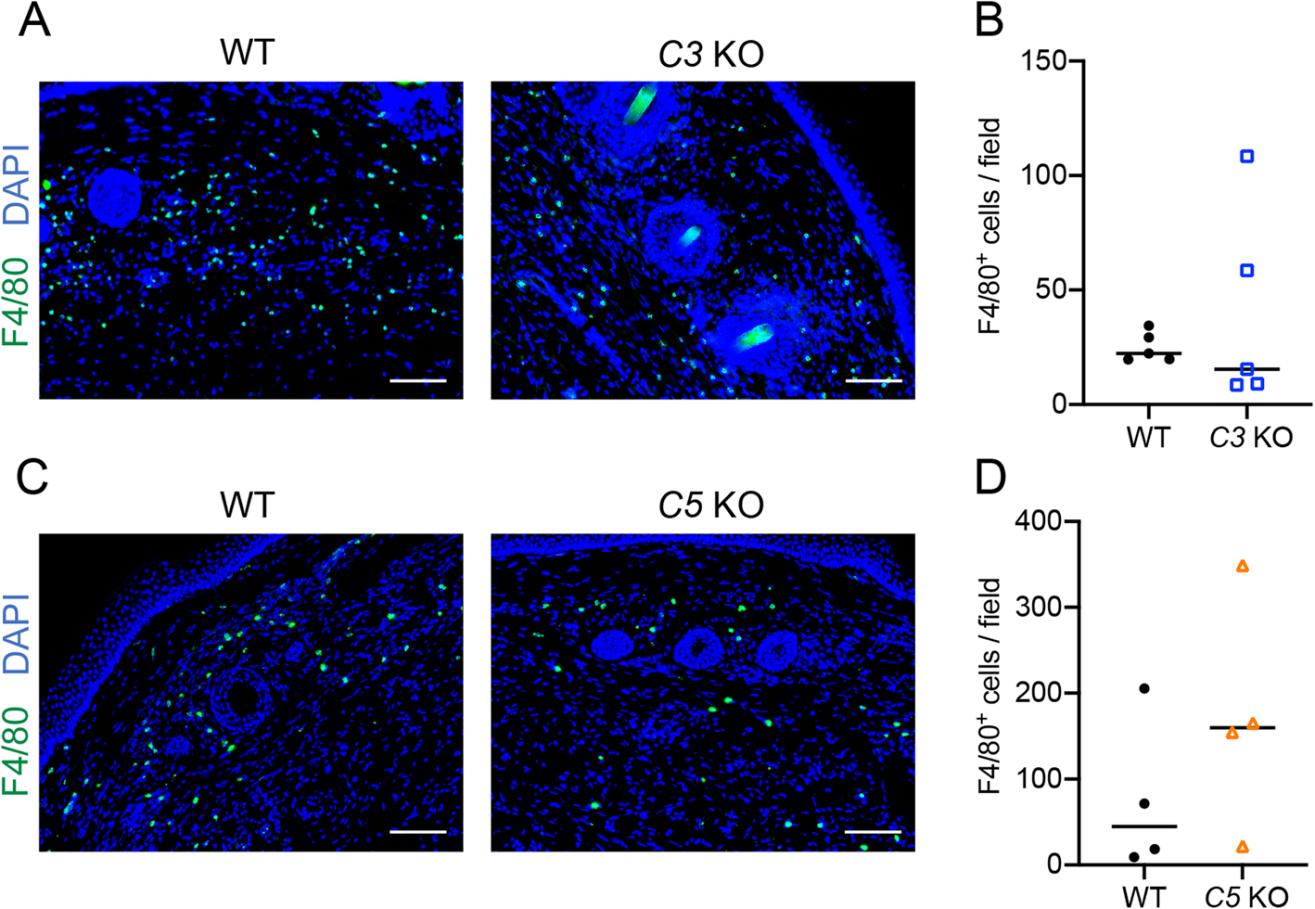
Infiltration of macrophages in lymphedema tissues on POD21. **A** Detection of F4/80^+^ macrophages (Green) in lymphedema tissues of wild-type (WT) and *C3* KO mice. **B** Comparison of numbers of F4/80^+^ macrophages per field (8 fields/mouse) of wild-type (WT) and *C3* KO mice in lymphedema tissues (*n* = 5/group). Horizontal bars indicate the averages. **C** Detection of F4/80^+^ macrophages (Green) in lymphedema tissues of wild-type (WT) and *C5* KO mice. **D** Comparison of numbers of F4/80^+^ macrophages per field (8 fields/mouse) of wild-type (WT) (*n* = 4) and *C5* KO (*n* = 5) mice in lymphedema tissues (*n* = 4/group). Horizontal bars indicate the averages. Representative images are shown in (**A**) and (**C**). Nuclei were stained with DAPI (Blue). Scale bars = 100 μm

To investigate whether macrophages and granulocytes infiltrate the lymphedema region in *C3* and *C5* KO mice models, we detected F4/80^+^ macrophages and Ly6G^+^ granulocytes using fluorescent immunohistochemistry. We detected increased infiltration of both F4/80^+^ macrophage cells (Fig. 3) and Ly6G^+^ granulocyte cells (Supplementary Fig. 4) into lymphedema tissues of some of *C3* KO and *C5* KO mice, but the change was not significant.

**Fig. 4:**
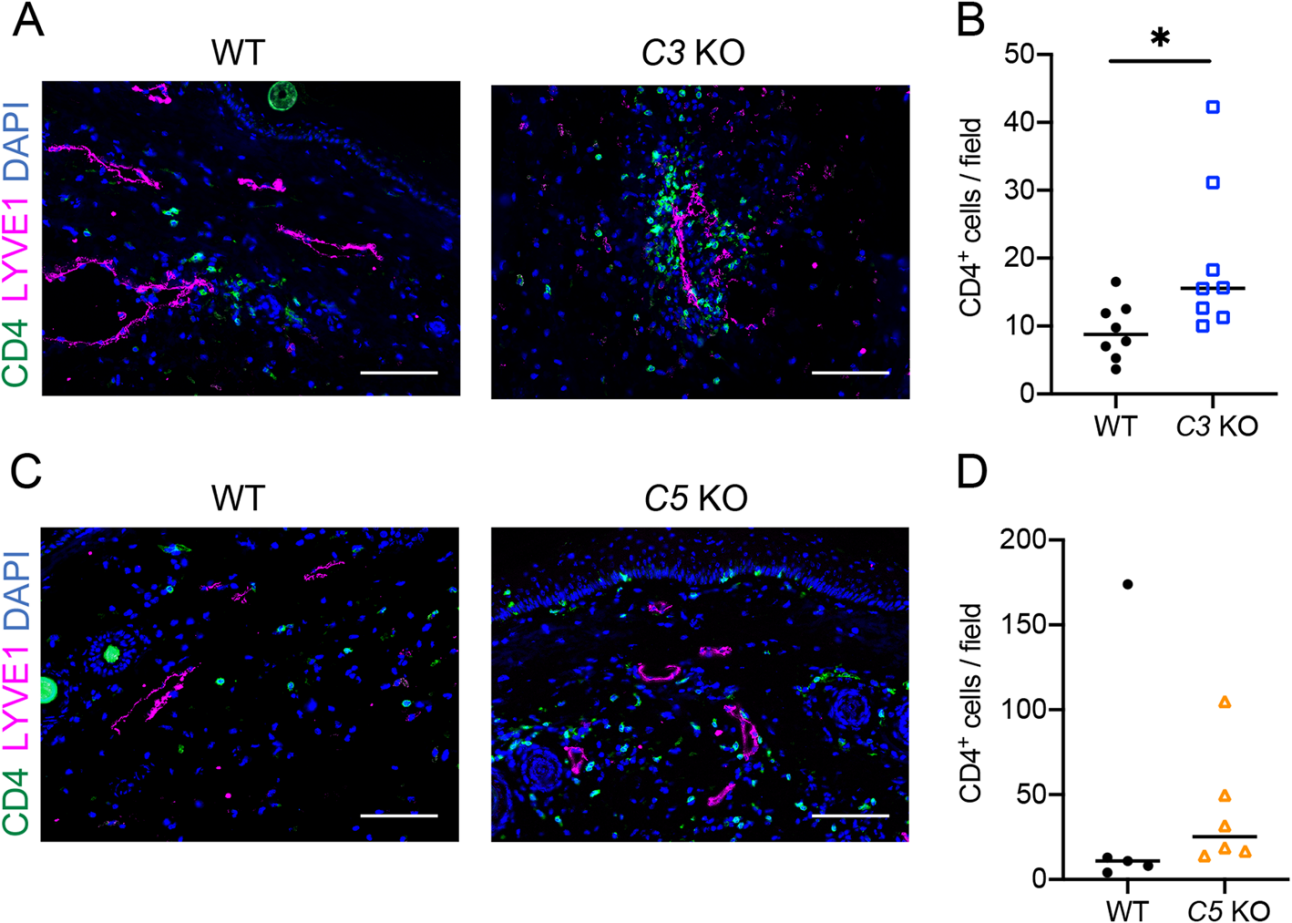
Infiltration of CD4^+^ T cells in lymphedema tissues on POD21.  **A** Detection of CD4^+^ T cells (Green) and lymphatic vessels (Magenta) in lymphedema tissues of WT and *C3* KO mice. **B** Comparison of numbers of CD4^+^ T cells per field (8 fields/mouse) of WT and *C3* KO mice in lymphedema tissues (*n* = 8/group). Horizontal bars indicate the averages; * *P*  < 0.05. **C** Detection of CD4^+^ T cells (Green) and lymphatic vessels (Magenta) in lymphedema tissues of WT and *C5* KO mice. **D** Comparison of numbers of CD4^+^ T cells per field (8 fields/mouse) of WT (*n* = 5) and *C5* KO (*n* = 6) mice in lymphedema tissues (*n* = 8/group). Horizontal bars indicate the averages.  Representative images are shown in (**A**) and (**C**). Nuclei were stained with DAPI (Blue). Scale bars = 100 μm

Next, we examined the infiltration of CD4^+^ T cells, which are known to be critical immune cells involved in the development of lymphedema. More CD4^+^ T cells infiltrated around lymphatic vessels and throughout lymphedema regions of *C3* KO mice compared with wild-type mice (Fig. 4A and B). We also investigated the infiltration of CD4^+^ T cells in regions of lymphedema of *C5* KO mice; the infiltration of CD4^+^ T cells into the lymphedema region tended to be increased in *C5* KO mice, but without statistical significance (Fig. 4C and D).

### Increased dead cells in lymphedema of ***C3 ***KO mice

**Fig. 5:**
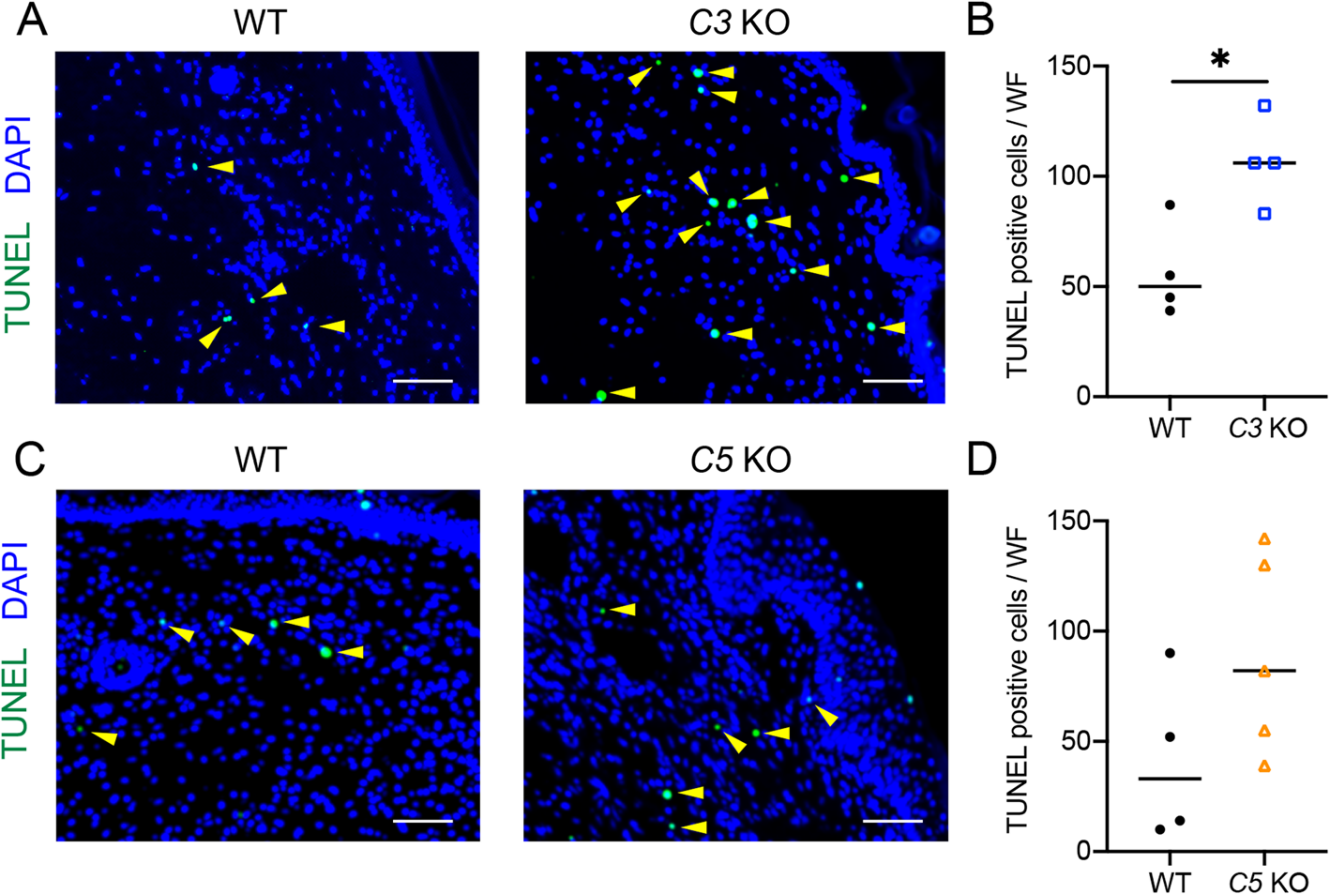
Accumulation of TUNEL^+^ cells in lymphedema tissues on POD21.  **A** Detection of TUNEL^+^ cells (Green) in lymphedema tissues of WT and *C3* KO mice.  **B** Comparison of number of TUNEL^+^ cells per whole field in lymphedema tissues of WT and *C3* KO mice (*n* = 4/group). Horizontal bars indicate the averages; * *P*  < 0.05. **C** Detection of TUNEL^+^ cells (Green) in lymphedema tissues of WT and *C5* KO mice. **D** Comparison of number of TUNEL^+^ cells per whole field in lymphedema tissues of WT (*n* = 4) and *C5* KO mice (*n* = 5). Horizontal bars indicate the averages. Representative images were shown in (**A**) and (**C**). Yellow arrowheads indicate TUNEL^+^ cells. Nuclei were stained with DAPI (Blue). Scale bars = 100 μm

Deficiency of *C3* causes impaired elimination of apoptotic and necrotic cells [18, 19], possibly resulting in the enhanced inflammation, so we used TUNEL analysis to examine the accumulation of dead cells in lymphedema regions (Fig. 5). TUNEL^+^ cells increased in the *C3* KO lymphedema region (Fig. 5A and B). Most of the TUNEL^+^ cells were appeared to be granulocytes as they were also positive for Ly6G (Supplementary Fig. 5). Cells with active caspase 3 were not detected in the *C3* KO lymphedema region. TUNEL^+^ cells in lymphedema regions are therefore indicated to be necrotic cells. TUNEL^+^ cells also tended to increase in the lymphedema region in some *C5* KO mice, but without significant difference (Fig. 5C and D).

## Discussion

In the present study, C3 was shown to be transcriptionally upregulated in the mouse tail lymphedema model and C3, as well as C4, was shown to be detected around the dilated lymph vessels. This suggests that lymphatic injury should induce activation of the classical or lectin pathway during lymphedema formation, leading to chronic inflammation. Chronic inflammation has been suggested to exacerbate lymphedema, and regulatory T cells (Treg) may inhibit the progression of lymphedema by suppressing inflammation [24]. Furthermore, the deficiency of *C3* or *C5* has been suggested in a mouse skin model to promote wound healing [17]. During the wound healing process, as well as the formation of lymphedema, inflammation is initially induced, which subsequently leads to local fibrosis of the skin. We therefore investigated the effects of the complement deficiency on the development of lymphedema. However, lymphedema of *C3* and *C5* KO mice showed no such improved phenotypes compared with wild-type mice as in wound healing. Interestingly, more immune cells seemed to infiltrate the lymphedema region in both *C3* KO and *C5* KO mice compared with in wild-type mice.

The complement system has an important role in eliminating altered host cells such as apoptotic or necrotic cells, as well as in protecting the host from pathogens [18, 19]. These altered host cells activate the classical pathway, and are opsonized by C3 degraded products. Macrophages recognize the opsonized cells via complement receptor 3 and phagocytose them. Impaired elimination of altered host cells by the complement system triggers various inflammatory responses, leading to autoimmune diseases such as systemic lupus erythematosus [25, 26]. In the present study, an increased number of dead cells was detected in the lymphedema region of *C3* KO mice compared with that of wild-type mice. Although without statistical significance, dead cells tended to be increased in the lymphedema of some *C5* KO mice. This may be because in *C5* KO mice, dead cells may be opsonized and removed by macrophages recruited by C3a, while in *C3* KO mice, there is no induction of opsonization of dead cells by C3 degradation products.

Activation of C3 and C5 induces the production of C3a and C5a, respectively, and these increase vascular permeability as anaphylatoxins and attract macrophages and granulocytes to the inflammatory sites. In some *C3* KO and *C5* KO mice, macrophages and granulocytes were not significantly increased in the lymphedema region, possibly because there was little or no production of C3a and C5a. However, the lack of a significant increase of dead cells in *C5* KO mice may be due to the production of C3a.

No prominent augmentation of edematous swelling was observed in *C3* KO mouse tails, but there was a significant increase in infiltration of CD4^+^ T cells in the lymphedema tissues. CD4^+^ T cells have been reported to increase in the tissue of lymphedema in a mouse lymph node-dissection model and in human lymphedema tissue after breast cancer surgery [13]. In the early stage of lymphedema, CD4^+^ T cells often activate macrophages to promote excessive lymphangiogenesis, leading to lymphedema [27]. CD4^+^ T cells could be sensitive to various kinds of tissue damage, such as increased debris and cell death [13]. Specifically, infiltration of Th2 CD4^+^ T cells characterized by signature cytokines such as IL-4, IL-5, and IL-13 promotes lymphedema but Treg cells inhibit the development of lymphedema. In the present study, a much larger number of TUNEL^+^ necrotic cells were observed in the lymphedema region of *C3* KO mice. The increased dead cells should trigger the accumulation of CD4^+^ T cells around lymphatic vessels and throughout lymphedema tissue. Although the activated complement and CD4^+^ T cells mainly accumulated around lymphatic vessels, the TUNEL^+^ cells were detected throughout the lymphedema tissue. Danger-associated molecular patterns derived from dead cells induce infiltration of CD4^+^ T cells, leading to chronic inflammation [28]. Future studies should analyze the molecular mechanism by which the impaired clearance of dead cells due to the complement deficiency leads to the infiltration of CD4^+^ T cells and effects of the complement deficiencies on the process of lymphedema through the enhanced inflammation.

## Conclusion

Complement-targeted therapies such as eculizumab and ravulizumab, anti-C5 antibodies, and sutimlimab, an anti-C1s antibody, have recently been applied for various hematologic, renal, and neuroimmune diseases. C2 deficiency and deficiencies of components of the terminal complement pathway including C5 are relatively common. Vigilance for exacerbation of lymphedema is required when surgical treatments are performed that may injure lymphatic vessels in patients undergoing these complement-targeted therapies, or in patients with complement deficiency.

## Materials and methods

### Mice

Wild-type C57BL/6J mice were purchased from CLEA Japan (Tokyo, Japan). *C3* KO mice (B6.129S4- C3tmlrr/J; #029661) were obtained from the Jackson Laboratory (Bar Harbor, ME) [29]. The *C3* KO mice and their control mice were male offspring of *C3* KO mice and wild-type mice, respectively, obtained by intercrossing *C3* heterozygous mice. *C5* KO mice were generated using the Alt-R CRISPR/Cas9 system (Integrated DNA Technologies, Inc.) (Supplementary Fig. 1A). The *C5* heterozygous mice were backcrossed to C57BL6/J mice more than five times. The *C5* KO mice and their control mice were male offspring of *C5* KO mice and wild-type mice, respectively, obtained by intercrossing the backcrossed mice. We used male wild-type and mutant mice as a tail lymphedema model because in preliminary experiments, there was great variation in the extent of female mouse lymphedema.

### Surgical model of lymphedema

A tail lymphedema model was created in 9 to 10-week-old male C56BL6/J and mutant mice, as previously described [1].

After the mice were fully anesthetized by intraperitoneal injection of mixed anesthetic agents [0.75 mg/kg medetomidine hydrochloride (Medetomin, Meiji Seika Pharma, Tokyo, Japan), 4 mg/kg midazolam (Dormicum, Astellas Pharma, Japan), 5 mg/kg butorphanol (Vetorphale, Meiji Seika Pharma, Tokyo, Japan)], a 3-mm wide strip of skin was circumferentially excised 1 cm distal to the base of the mouse tail, and lymphatic vessels in the superficial skin layer were removed. After patent blue was injected subcutaneously 2 cm distal to the surgical site of the mouse tail, the deep lymphatic vessels running parallel to the lateral tail vein were ligated with 10 − 0 nylon (Supplementary Fig. 2A and B). After the surgical treatment, the mice were intraperitoneally treated with 0.75 mg/kg atipamezole (Mepatia, Meiji Seika Pharma, Tokyo, Japan). The operated tail region was circumferentially wrapped with an Opsite Quick Roll (Smith & Nephew, Watford, UK) to keep it moist for 24 h. To obtain tissues for RNA and histological analysis, the mice were euthanized by cervical dislocation.

## Measurement of tail swelling volumes

Mouse tails after surgery were digitally photographed on a weekly basis, and the tail volumes were calculated using the following truncated cone formula (Supplementary Fig. 2C) [30] :

$$\varvec{V}=\frac{5}{12}\varvec{\pi }\left\{\left(\varvec{R}{1}^{2}+\varvec{R}1\varvec{R}2+\varvec{R}{2}^{2}\right)+\left(\varvec{R}{2}^{2}+\varvec{R}2\varvec{R}3+\varvec{R}{3}^{2}\right)\dots +\left(\varvec{R}{8}^{2}+\varvec{R}8\varvec{R}9+\varvec{R}{9}^{2}\right)\right\}$$

Tail diameters (***R1, R2***,…and ***R9***) were measured with ImageJ (public domain, developed by Wayne Rasband at the National Institutes of Health) at every 5 mm interval from 2 to 42 mm distal to the mouse tail surgical site [31].

### Real-time qPCR analysis

Total RNA was extracted from the lymphedema region of the mouse tail using RNeasy Mini Kit (Qiagen, Venlo, Netherlands) according to the manufacturer’s protocol. Bone-depleted mouse tail tissues were disrupted in buffer RLT by using a multi-beads shocker (Yasui Kikai, Osaka, Japan). cDNA was synthesized using SuperScript III First-Strand Synthesis System (Thermo Fisher Scientific, Waltham, MA). Real-time qPCR was performed by the TaqMan gene expression assay with TaqMan Primer and probe sets for *C3* (Mm01232779_m1, Thermo Fisher Scientific, Waltham, MA) and 18 S ribosomal RNA probe as the internal control (Hs99999901_s1, Thermo Fisher Scientific). We determined the threshold cycle (Ct) to the relative standard curve method to calculate the relative quantification of mRNA expression.

### Histology and fluorescent immunohistochemistry

Lymphedema tail tissues were fixed in 4% paraformaldehyde at 4 °C for 48 h, decalcified using 10% ethylenediaminetetraacetic acid in phosphate buffered saline (PBS), and embedded in paraffin. Paraffin sections were prepared at 6 μm, and stained with hematoxylin and eosin (H&E) or specific antibodies (Supplementary Table 1). Frozen tissues were cryoprotected with 30% sucrose/PBS after decalcification, and embedded in OCT compound (Sakura Finetek, Tokyo, Japan) and 30% sucrose/PBS. Frozen sections were prepared at 18 μm.

For immunofluorescent staining, antigen retrieval was achieved by sodium citrate (pH 6.0) or Tris-EDTA (pH 9.0) at 121 °C, for 1 min using an autoclave. Sections were incubated at 4 °C overnight with primary antibodies (Supplementary Table 1) and appropriate secondary antibodies conjugated with Alexa Fluor 488 and 568 for 1 h at room temperature. Vector TrueVIEW Autofluorescence Quenching Kit with DAPI (Vector Laboratories, Newark, CA) was used to reduce erythrocyte autofluorescence.

The TUNEL method was performed using Apoptag Fluorescein in Situ Apoptosis Detection Kit (Sigma-Aldrich, St. Louis, MO) and nuclei were visualized with DAPI. Images of sections were taken using BZ X-800 microscope (Keyence, Osaka, Japan) and analyzed using ImageJ.

### Statistical analysis

Student’s T-test was used to compare differences between the two groups. *P* < 0.05 was considered to indicate a significant difference.

### Supplementary Information

**Additional file 1. **The full length original images in Supplementary Fig. 1.

**Additional file 2. Supplementary Fig. 1.** Establishment of *C5* knockout (KO) mice. **Supplementary Fig. 2.** Surgical model of lymphedema. **Supplementary Fig. 3.** Representative pictures of tail lymphedema on POD21 in wild-type (WT), *C3* KO and *C5* KO mice. **Supplementary Fig. 4.** Infiltration of granulocytes in lymphedema tissues on POD21. **Supplementary Fig. 5.** Detection of TUNEL^+^ granulocytes in lymphedema tissues on POD21. **Supplementary Table 1.** List of antibodies.

## Data Availability

The datasets used and/or analyzed during the current study are available from the corresponding author upon reasonable request.

## Abbreviations

KO: knockout
C5aR1: C5a receptor 1
MAC: membrane attack complex
PNH: paroxysmal nocturnal hemoglobinuria
POD: postoperative date
Treg: regulatory T cell
Ct: threshold cycle
PBS: phosphate buffered saline
H&E: hematoxylin and eosin
