# Supplementary material for: Increased infiltration of CD4+ T cell in the complement deficient lymphedema model
Source: BMC Immunol. 2023 Nov 8;24:42. doi: 10.1186/s12865-023-00580-1 (PMC10633916; doi:10.1186/s12865-023-00580-1)
Supplement: Supplementary file 1 — Additional file 1. The full length original images in Supplementary Fig. 1. [file 12865_2023_580_MOESM1_ESM.docx]

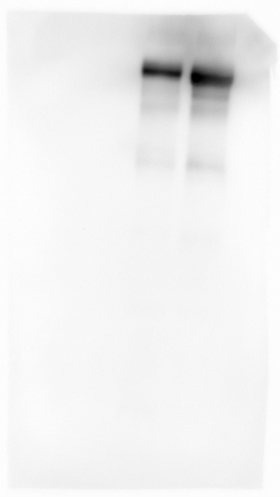

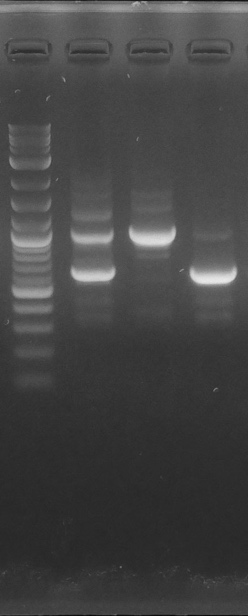

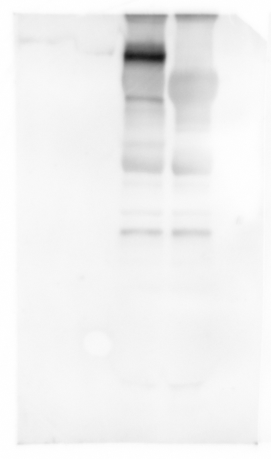
A B C

M

Het

KO

WT

KO

WT

KO

WT

**Additional File 1** The full length original images in Supplementary Fig. 1

1. Genetic analysis of heterozygous (Het), wild-type (WT) and *C5* KO mice by PCR. M indicates size-marker (2-Log DNA ladder, New England Biolabs, Ipswich, MA).
2. Expression of C5 protein in 1 mL serum of wild-type (WT) and *C5* KO mice.
3. Expression of IgG protein in 0.2 mL serum of wild-type (WT) and *C5* KO mice.
