# Supplementary material for: Increased infiltration of CD4+ T cell in the complement deficient lymphedema model
Source: BMC Immunol. 2023 Nov 8;24:42. doi: 10.1186/s12865-023-00580-1 (PMC10633916; doi:10.1186/s12865-023-00580-1)
Supplement: Supplementary file 2 — Additional file 2. Supplementary Fig. 1. Establishment of C5 knockout (KO) mice. Supplementary Fig. 2. Surgical model of lymphedema. Supplementary Fig. 3. Representative pictures of tail lymphedema on POD21 in wild-type (WT), C3 KO and C5 KO mice. Supplementary Fig. 4. Infiltration of granulocytes in lymphedema tissues on POD21. Supplementary Fig. 5. Detection of TUNEL+ granulocytes in lymphedema tissues on POD21. Supplementary Table 1. List of antibodies. [file 12865_2023_580_MOESM2_ESM.docx]

**Supplementary Material**

**Supplementary Materials and Methods**

**Mice**

*C5* deficient mice (*C5* KO) were generated by using the Alt-R CRISPR/Cas9 system (Integrated DNA Technologies, Inc.). Briefly, crRNAs for the first intron sequence: 5-AGCTCACTACCTCTGCATGAAGG-3’ (gRNA1) and the second intron sequence: 5’-TAGCGTTCTTCTACAACTGTGGG-3’ (gRNA2) were annealed with tracrRNA and then incubated with Cas9 nuclease to gain the crRNA/tracrRNA-Cas9 complex, according to the manufacturer’s instructions. The complex (final each 3.3 µM crRNA; 5 µM tracrRNA; 1 µM Cas9) was transferred into 120 C57BL/6N pronuclear stage embryos by electroporation (NEPA21 electroporator, Nepa Gene, Ichikawa, Japan), and the following day, 80 of two cell stage embryos were transplanted into four pseudo-pregnant mice. Offspring mice were subjected to genotyping with PCR and sequence analysis. *C5* KO mice had 404 bp deletion containing exon 2.

Mice were genotyped by using the following primers: 5’-ATCTTGAGTGCACCAAGCC-3’, 5’-GGTTGCAGCAGTCTATGAAGG-3’ and 5’-GCCAGAGGCCACTTGTGTAG-3’ for *C3*KO, and 5’-GAGGTTCCACTGCGGTCTTTCTA-3’ and 5’-AGCATCCAGCAATATACTACAGGTCA-3’ for *C5*KO.

**Western blotting**

The mouse serum (1 mL for C5 or 0.2 mL for IgG) were separated by sodium dodecyl sulfate polyacrylamide gel electrophoresis under non-reducing condition and transferred to Immobilon-P PVDF membranes (MilliporeSigma, Burlington, MA). The expression of C5 was incubated with goat anti-human C5 antibody in Can Get Signal Immunoreaction Enhancer Solution 1 (Toyobo, Osaka, Japan) and with peroxidase-conjugated AffiniPure Donkey anti-Goat IgG (H+L) in Can Get Signal Immunoreaction Enhancer Solution 2 (Toyobo, Osaka, Japan), and visualized using the ECL Prime Western Blotting System (GE Healthcare, Buckinghamshire, England). The expression of IgG as a control was detected with horseradish peroxidase-anti-mouse IgG in Can Get Signal Immunoreaction Enhancer Solution 1 (Toyobo, Osaka, Japan). All antibodies are listed in Supplementary Table 1. Proteins on membranes were detected using LuminoGraph (ATTO, Amherst, NY).

**Supplementary Figures**

**Supplementary Fig. 1** Establishment of *C5* knockout (KO) mice

(A) Structure of *C5* wild-type and knockout alleles. Two gRNAs (black arrowheads, gRNA1 and 2) were designed in the intron 1 and 2 of the *C5* gene. The *C5* KO allele has 404 bp deletion including exon 2 of the *C5* gene. Primer 1 and 2 (black arrows) were designed for the genotyping.

(B) *C5* KO (KO) and wild-type (WT) mice were distinguished by PCR analysis with primer 1 and 2. Wild-type and KO alleles are indicated by 1 and 2 arrows, respectively.

(C) Expression of C5 protein in serum of wild-type (WT) and *C5* KO mice. Serum was loaded at non-reduced condition. IgG in serum was detected as a loading control.

The full-length images of (B) and (C) are shown in Additional file 1.

**Supplementary Fig. 2** Surgical model of lymphedema

(A) Illustration for the tail skin excision to generate lymphedema. Black arrows indicate deep lymphatic vessels (Blue). Veins and arteries are shown by red and pink, respectively.

(B) Deep lymphatic vessels are stained with patent blue dye (black arrows). These lymphatic vessels were ligated to generate lymphedema.

(C) Representative pictures of tails on postoperative day 0 (POD0) and 21 (POD21).

The tail volume is calculated using the truncated cone formula. Lengths of yellow lines (*R1*, *R2*,…and *R9*) every 5 mm from distal of the surgical site (white arrow) were measured as tail diameters.


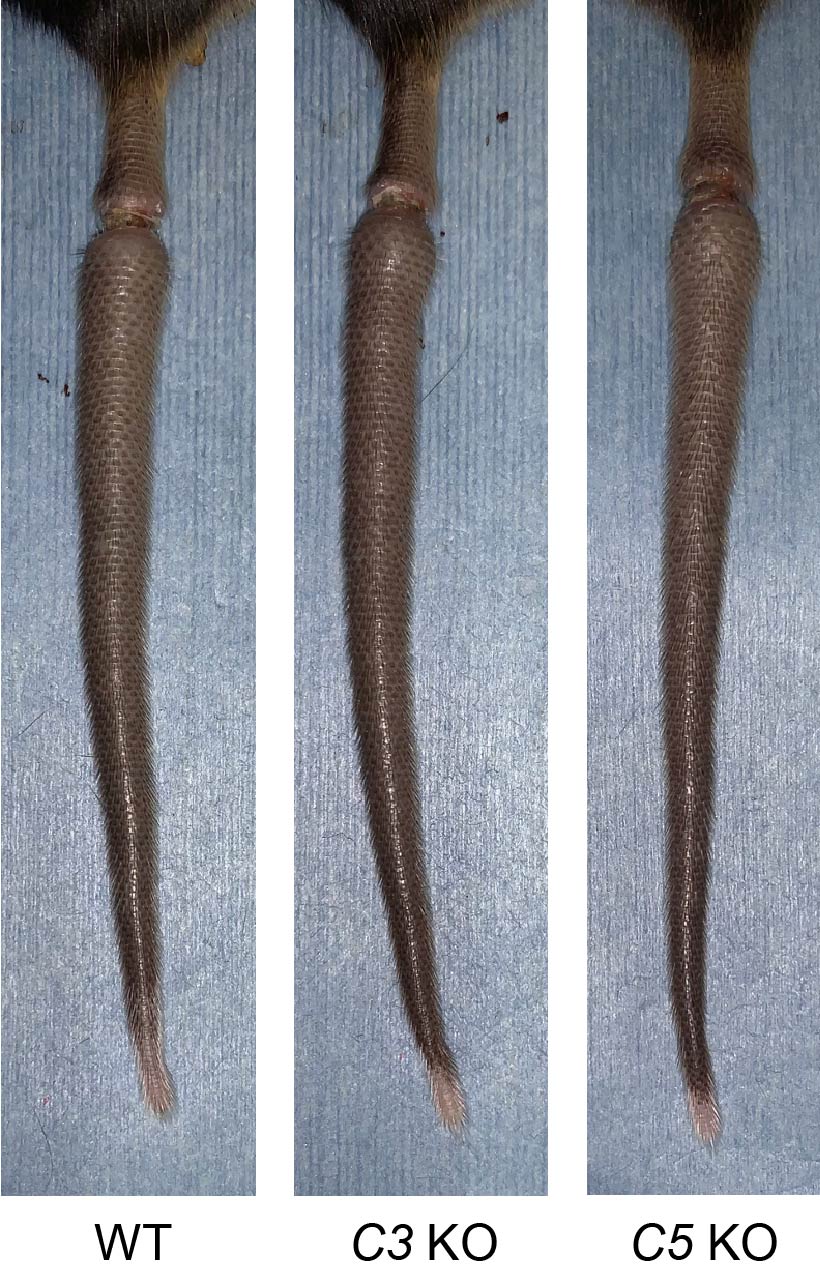


**Supplementary Fig. 3** Representative pictures of tail lymphedema on POD21 in wild-type (WT), *C3* KO and *C5* KO mice.


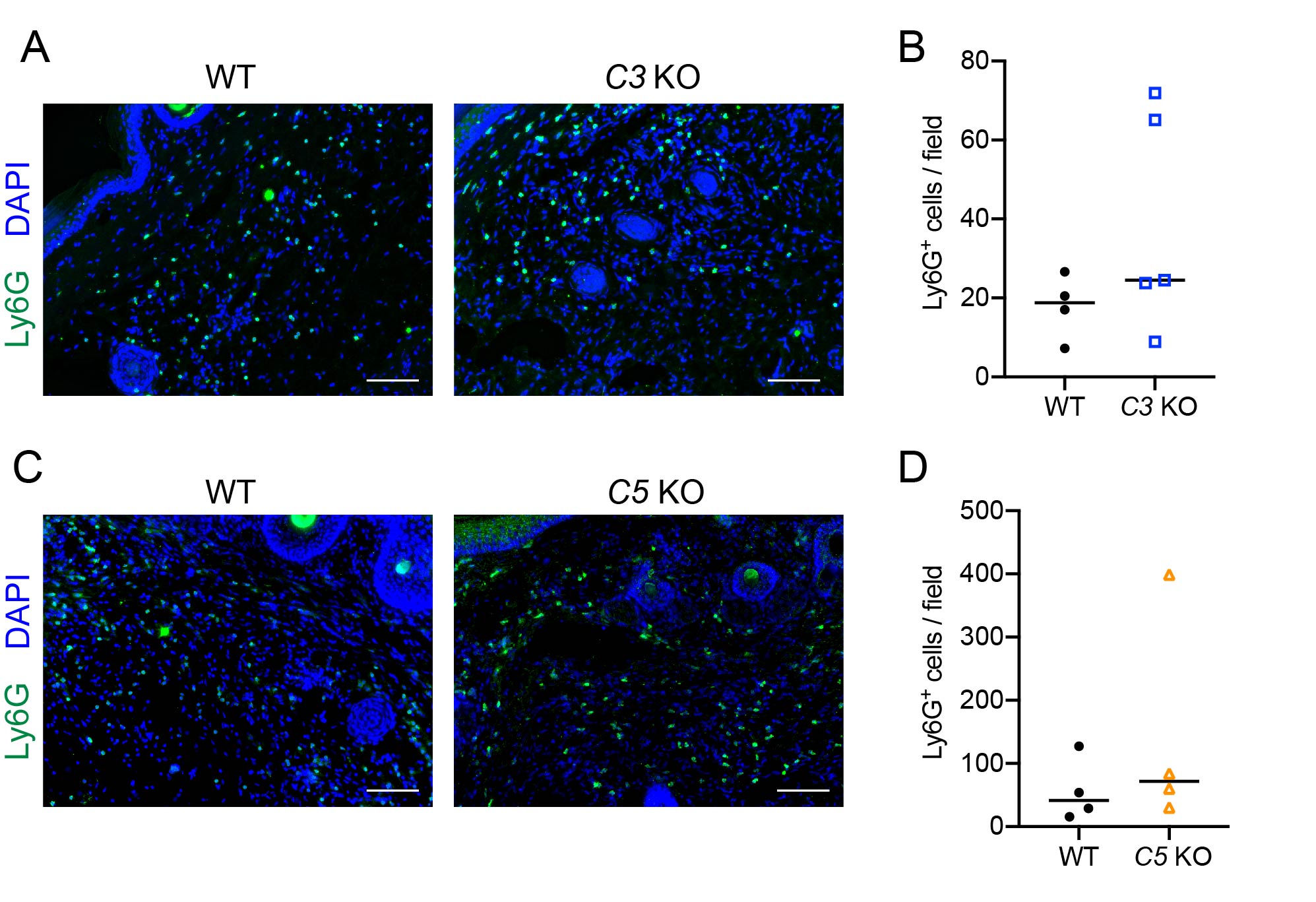


**Supplementary Fig. 4** Infiltration of granulocytes in lymphedema tissues on POD21.

(A) Detection of Ly6G^+^ granulocytes (Green) in lymphedema tissues of wild-type (WT) and *C3* KO mice.

(B) Comparison of numbers of Ly6G^+^ granulocytes per field (8 fields/mouse) of wild-type (WT) (n=4) and *C3* KO (n=5) mice in lymphedema tissues. Horizontal bars indicate the averages.

(C) Detection of Ly6G^+^ granulocytes (Green) in lymphedema tissues of wild-type (WT) and *C5* KO mice.

(D) Comparison of numbers of Ly6G^+^ granulocytes per field (8 fields/mouse) of wild-type (WT) and *C5* KO mice in lymphedema tissues (n=4/group). Horizontal bars indicate the averages.

Representative images are shown in (A) and (C). Nuclei were stained with DAPI (Blue). Scale bars = 100 μm.

**
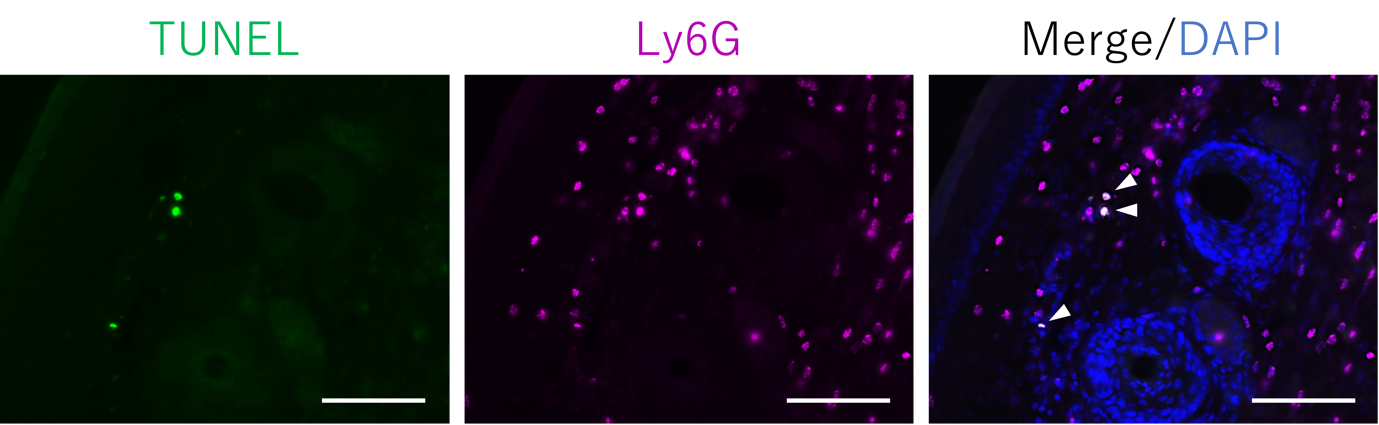
**

**Supplementary Fig. 5** Detection of TUNEL^+^ granulocytes in lymphedema tissues on POD21.

TUNEL^+^ cells (Green) were detected in some Ly6G^+^ granulocytes (Magenta) in lymphedema tissues on POD21. Representative images (*C5* KO mice) are shown. White arrowheads indicate TUNEL^+^ granulocytes. Nuclei were stained with DAPI (Bule). Scale bars = 100 μm.

**Supplementary Table 1** List of antibodies

| Antibody | Application | Host Animal | Dilution | Distributor | Cat. No., RRID |
| --- | --- | --- | --- | --- | --- |
| Anti-LYVE1 | IHC-P | goat,  polyclonal | 1:200 | R&D Systems,  Minneapolis, MN, USA | #AF2125,  RRID: AB_2297188 |
| Anti-C3 | IHC-P | rabbit,  polyclonal | 1:50 | Hycult Biotech,  Uden, Netherlands | #HP8012,  RRID: AB_10130523 |
| Anti-C4d | IHC-P | rabbit,  polyclonal | 1:50 | Hycult Biotech,  Uden, Netherlands | #HP8033,  RRID: AB_10681053 |
| Anti-F4/80 | IHC-F | rabbit,  monoclonal | 1:500 | Cell Signaling Technology,  Danvers, MA, USA | #70076,  RRID: AB_2799771 |
| Anti-Ly-6G | IHC-F | rat,  polyclonal | 1:400 | Biolegend,  San Diego, CA, USA | #127601,  RRID: AB_1089179 |
| Anti-CD4 | IHC-P | rabbit,  monoclonal | 1:1000 | Abcam,  Cambridge, UK | #ab183685,  RRID: AB_2686917 |
| Alexa Fluor 568-  anti-Goat IgG | IHC-P, F | donkey,  polyclonal | 1:400 | Thermo Fisher Scientific,  Waltham, MA, USA | #A-11057,  RRID: AB_2534104 |
| Alexa Fluor 488-  anti-Rabbit IgG | IHC-P, F | donkey,  polyclonal | 1:400 | Thermo Fisher Scientific,  Waltham, MA, USA | #A-21206,  RRID: AB_2535792 |
| Alexa Fluor 488-  anti-Rat IgG | IHC-P, F | donkey,  polyclonal | 1:400 | Thermo Fisher Scientific,  Waltham, MA, USA | #A-21208,  RRID: AB_2535794 |
| Alexa Fluor 568-  anti-Rat IgG | IHC-F | donkey,  polyclonal | 1:400 | Thermo Fisher Scientific,  Waltham, MA, USA | #A78946  RRID: AB_2910653 |
| Anti-Human C5 | WB | goat,  polyclonal | 1:1000 | Quidel,  San Diego, CA, USA | #A306,  RRID: AB_452507 |
| HRP-  anti-Goat IgG | WB | donkey,  polyclonal | 1:10000 | Jackson ImmunoResearch Labs,  West Grove, PA, USA | #705-035-147,  RRID: AB_2313587 |
| HRP-  anti-mouse IgG | WB | horse,  polyclonal | 1:10000 | Cell Signaling Technology,  Danvers, MA, USA | #7076,  RRID:AB_330924 |

IHC-P: Immunohistochemistry-Paraffin, IHC-F: Immunohistochemistry-Frozen, WB: Western Blot

HRP: Horseradish Peroxidase
